# Supplementary material for: Complexome profiling of the Chlamydomonas psb28 mutant reveals TEF5 as an early PSII assembly factor
Source: Plant Cell. 2025 Mar 17;37(6):koaf055. doi: 10.1093/plcell/koaf055 (PMC12164590; doi:10.1093/plcell/koaf055)
Supplement: koaf055_Supplementary_Data [file koaf055_supplementary_data.zip › psb28_interactive.zip!/plots/intensity/qualityPlots/peptides/_Cre11.g467773.t1.1.html]

### Cre11.g467773.t1.1

Trivial name:   
SeqCoverage [median]: 27.90  
Mass: 20.34 kDa  
  
 

| Sequence | MissedCleavage | Mass [Da] | wtIntensity | muIntensity | Score |
| --- | --- | --- | --- | --- | --- |
| AAISQVAINIYK | 0 | 1289.73 | 0.00 | 1583.26 | 116.30 |
| AGADVNEREGGADGDTPLLMIAR | 1 | 2327.12 | 0.00 | 906.38 | 60.03 |
| SGASTAGVASIK | 0 | 1047.56 | 0.00 | 589.72 | 79.15 |
|  |  |  | 0.0 | 3079.4 |  |
